# Case report: caught by the pill cam…literally

**DOI:** 10.1093/jscr/rjac353

**Published:** 2022-07-30

**Authors:** Dylan S Goto, Christina J Wai

**Affiliations:** Department of Surgery, John A. Burns School of Medicine, University of Hawaii, Honolulu, HI, USA; Department of Surgery, Surgical Oncology, John A. Burns School of Medicine, University of Hawaii, Honolulu, HI, USA

## Abstract

Small bowel obstructions are a common general surgery occurrence. In a patient with prior abdominal surgeries, the usual diagnosis is secondary to adhesions. The management is typically conservative, which usually avoids operative intervention. Computed tomography (CT) scans help diagnosticians take a snapshot inside the abdomen; however, CT scans are not perfect and intra-abdominal pathologies can be missed requiring surgery. We present a case of an atypical small bowel obstruction. The initial CT scan showed a transition point in the right lower quadrant, which was managed non-operatively. One week later, the patient then re-presented with hematochezia following an outpatient pill cam procedure. Imaging showed the presence of a new small bowel mass, which was not seen on imaging done 1 week ago or from 10 months prior. He was then taken for a diagnostic laparoscopy, in which a small bowel mass was found, pathology positive for recurrent renal cell carcinoma.

## INTRODUCTION

It is estimated that small bowel obstructions account for ~12–16% of surgical admissions, resulting in 300 000 annual operations in the USA [1]. It is caused either by a functional (ex. ileus) or mechanical source. Post-operative adhesions remain to be the most common cause of small bowel obstructions at about 70%, followed by hernias, malignancies or inflammatory bowel disorders [2]. These adhesions are typically due to a history of prior abdominal surgery [3]. They are commonly diagnosed with computed tomography (CT) scans with intravenous (IV) contrast unless it is contraindicated, with a diagnostic accuracy >90% [4, 5]. They have the added benefit of differentiating the underlying etiologies such as transition points, hernias or masses that may be missed on an X-ray. It also helps to distinguish possible complications such as ischemia, perforation or hemorrhage, which can help to direct therapeutic management.

Small bowel obstructions are typically managed with bowel rest, nasogastric tube decompression and fluids. Obstructions secondary to adhesions are successfully managed non-operatively about 70–80% of the time [6, 7]. The administration of hypertonic water-soluble contrast, such as gastrografin, has been used to reduce the time to resolution, reduce the hospital length of stay and further evaluate the need for surgical intervention [8, 9]. Those that fail to resolve with gastrografin or show signs of bowel compromise proceed to surgery.

## CASE REPORT

**Figure 1:**
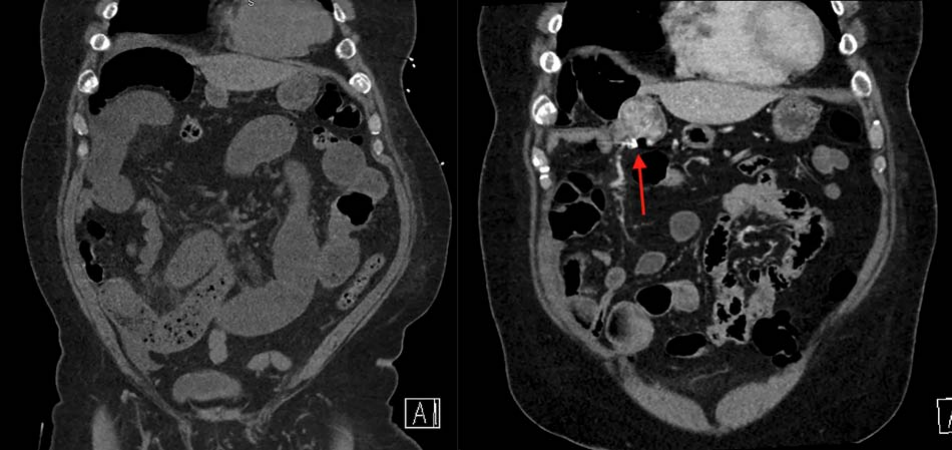
On the left is the initial CT scan non-contrast demonstrating dilated small bowel loops and a transition point in the right lower quadrant. On the right is the CT angiography (CTA), performed a week later, showing a mass and pill cam in the right upper quadrant.

**Figure 2:**
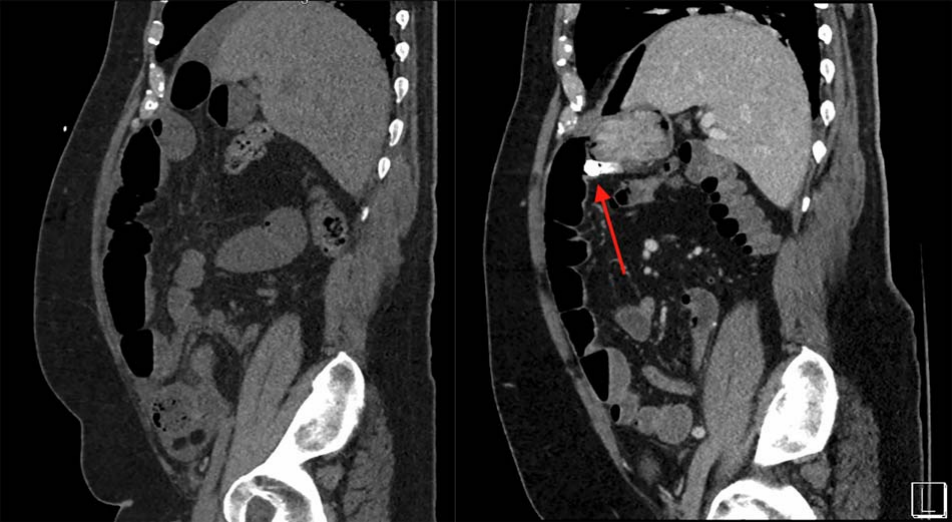
On the left is the initial CT scan non-contrast demonstrating no obvious mass. On the left is the CTA, a week later, showing a mass and pill cam in the right upper quadrant.

The patient is a 69-year-old male with a past medical history of hypertension, chronic kidney disease, chronic obstructive pulmonary disease, right renal cancer status post-laparoscopic nephrectomy in 2016 (pathology showed renal cell carcinoma with invasion into the adrenal gland, Fuhrman nuclear grade 3–4, pT4 pN0, Stage IV) who presented to the emergency room (ER) with abdominal pain. The patient recently underwent an esophagogastroduodenoscopy and a colonoscopy the week prior as a workup for anemia, which were unremarkable. Since then, he started having intermittent abdominal pain and bloating. The CT abdomen and pelvis without contrast in the ER showed multiple dilated small bowel loops with a transition point in the right lower quadrant (Figs 1 and 2). Due to his history of previous abdominal surgery, this was diagnosed as a small bowel obstruction secondary to adhesions. He was initially managed with a nasogastric tube and gastrografin study. On hospital Day 1, he had minimal output from his nasogastric tube and started to have bowel movements; therefore, his nasogastric tube was removed and he was started on a liquid diet. His diet was advanced, and on hospital Day 2, the patient was discharged.

The next week, unbeknownst to his Gastroenterologist about his recent small bowel obstruction admission, he then proceeded with a capsule endoscopy study to assess for other causes of his anemia. He tolerated the pre-operative bowel preparation without issues. After swallowing the capsule, he subsequently had bloody bowel movements. He then went to the ER and found to have a hemoglobin of 7.1, which was decreased from his prior week’s hospitalization, 8.4. A CT angiography (CTA) abdomen and pelvis was then performed that showed a 4 × 6 × 4.1 cm mass in the right upper quadrant with the capsule endoscopy at the level of the mass (Figs 1 and 2). The images from the capsule were unremarkable. On review of his imaging with radiology, it appeared that this mass was not apparent without IV contrast on his previous CT scan a week prior. In addition, a prior CT abdomen pelvis with IV contrast 10 months ago was negative for a small bowel mass. A repeat abdominal and pelvis CT with oral and IV contrast was done for confirmation. This re-demonstrated the 6 cm mass with the capsule stuck proximal to it; therefore, we performed a diagnostic laparoscopy.

**Figure 3:**
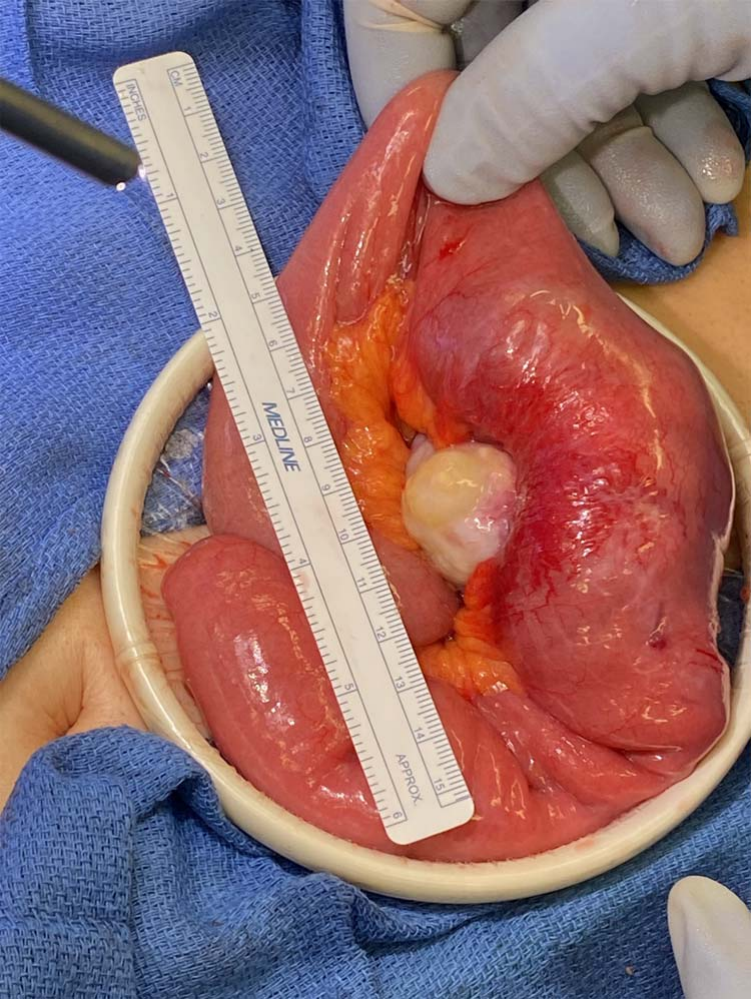
Small bowel mass found during the diagnostic laparoscopy.

**Figure 4:**
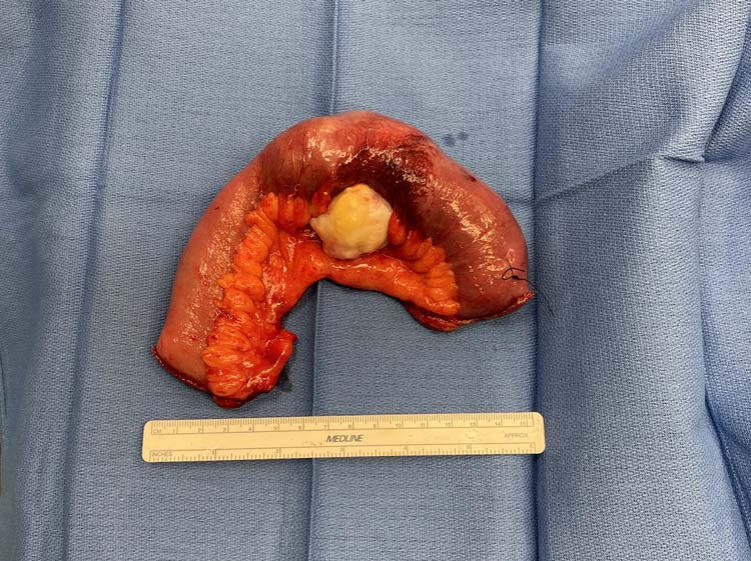
Small bowel mass specimen, post-resection.

Surgery revealed a small bowel tumor in the jejunum (Figs 3 and 4), which was resected and intestines re-anastomosed. The capsule was not identified within the lumen of the resected segment and had traveled through the obstructed segment distally. He had an unremarkable recovery and sequential X-rays demonstrated passage of the capsule. The pathology revealed metastatic renal cell carcinoma with intraluminal mucosal ulceration, 0/6 lymph nodes positive. He did well post-operatively and was referred back to his oncologist for further evaluation of his cancer.

## DISCUSSION

According to the Surveillance, Epidemiology, and End Results database, the 5-year survival of stage IV renal cell carcinoma is 12% [10]. Renal cell carcinoma has a high recurrence rate within the first 5 years even following nephrectomy. The recurrence rates following nephrectomy have been reported to be 7% for T1 tumors with a median interval of 38 months, 26% for T2 tumors with a median interval of 32 months and 39% for T3 tumors with a time interval of 17 months [11]. The most common locations of metastasis are spread to the lungs, followed by spread to the bone, liver, brain and local regional recurrence. It is estimated that local recurrence ranges from 1.8 to 27% [11]. Therefore, follow-up surveillance is highly recommended.

Surveillance protocol varies by the American Urologic Association and the National Comprehensive Cancer Network. Based on this patient’s staging, both guidelines recommend follow-up CT chest imaging, as well as CT, magnetic resonance imaging or ultrasound of the abdomen every 3–6 months until 5 years [12, 13]. Additional brain or bone imaging is recommended based on symptoms or clinical judgment. New biomarkers are being developed to help aid in narrowing patients that are at high risk of recurrence, which may help to tailor future surveillance protocols [14, 15].

This is a rare case of a metastatic renal cell carcinoma leading to anemia and a small bowel obstruction that was identified after a capsule endoscopy procedure. Prior non-contrast CT a week prior and another CT with IV contrast 10 months prior were negative for a small bowel mass. Therefore, this 6 cm mass remained undetected by prior imaging and was only found after he became symptomatic from the capsule itself. Even the capsule images were ineffective. CT scans have a high rate of sensitivity and specificity, but are not flawless. When adhesive disease is not the cause of a bowel obstruction, it is important to look for other possible etiologies. As medicine continues to advance, new strategies for monitoring kidney cancer recurrence are on the horizon.
